# The Use of Green Laser in LiDAR Bathymetry: State of the Art and Recent Advancements

**DOI:** 10.3390/s23010292

**Published:** 2022-12-27

**Authors:** Anna Szafarczyk, Cezary Toś

**Affiliations:** Faculty of Environmental and Power Engineering, Cracow University of Technology, Warszawska St. 24, 31-155 Cracow, Poland

**Keywords:** green laser, LiDAR bathymetry, coastal zones, Secchi depth, fluvial processes, abrasion, bottom surface morphology

## Abstract

Bathymetric LiDAR technology is a technology used for simultaneous data acquisition regarding the morphology of the bottom of water reservoirs and the surrounding coastal zone, realized from the air, e.g., by plane or drone. Contrary to the air topographic LiDAR, which uses an infrared wavelength of 1064 nm, bathymetric LiDAR systems additionally use a green wavelength of 532 nm. The green laser can penetrate the water, which makes it possible to measure the depth of shallow water reservoirs, rivers, and coastal sea waters within three Secchi depths. This article presents the theoretical basis for the construction of a green laser. Against the background of other methods of measuring the bottom of water reservoirs, the technology using waves from the visible light range is presented in detail in the assessment of the bottom morphology of shallow water reservoirs. The possibilities of using green laser in lidar bathymetry implemented in particular in non-navigable regions are shown. The results of the researchers’ work on river processes (erosion, sedimentation), design of stream restoration, determination of morphometric parameters of the riverbed, as well as assessment of the topography of the marine coastal bottom zones are summarized. The development direction of lidar bathymetry is discussed.

## 1. Introduction

In recent years, there has been considerable interest in mapping shallow inland water bodies [1,2] and marine coastal zones [3] due to statutory provisions [4] obliging Member States to document their freshwater resources and to take measures to protect their waters. Moreover, this obligation should be performed additionally after flood events or in the case of changes made by man in the flow system. A global and detailed description of the shape of the riverbed [5] is necessary for the implementation of many tasks in the field of hydrology, hydromorphology, hydrobiology, and hydraulics, such as flood simulation, sediment transport modeling, and habitat mapping [6].

Due to climate change and the constant rise in sea levels as a result of global warming, coastal zone management is of particular importance and needs to be monitored [7]. All spatial planning and coastal activities as well as offshore hydrological research and coastal engineering applications require bathymetric information [8,9,10].

Modeling the bottom of a water reservoir and the surface of the surrounding land is possible with the use of many direct and remote measurement technologies [11]. These include: depth measuring instruments (handheld probes, stick probes, gauge poles) and remote measurement systems such as: hydroacoustic systems: single and multi-beam echosounders, multi-transducer echosounders or bathymetric interferometric systems that measure from a boat, as well as aerial and satellite laser systems. The basic features of the sounding methods are presented in Table 1, where they are compared with the system using a green laser.

Until the use of LiDAR bathymetry, the results of the measurements of coastal zones and the shape of the bottom of the water reservoir had to be compiled together into one data set, in which the data were not uniform. The discrepancy in the data resulted from such factors as: work in various coordinate systems, characteristic for a given device/sensor, changes in morphology resulting from a time shift in the implementation of measurements performed with two different devices [12]. The solution to these inconveniences is the use of one device acquiring data for the water reservoir and the surrounding area at the same time. The accuracy of obtaining uniform data may then be lower, and the problem of the time shift between the performed measurements does not exist. Such a measurement technology is LiDAR bathymetry [13], which is a combination of the use of infrared light with a wavelength of 1064 nm, with a laser emitting green light with a wavelength of 532 nm [14]. It is a cost-effective solution for mapping the environment over large land and coastal zones to simulate flooding. Automated acquisition of map data on territorial and marine waters allows for the detection of changes and possible decisions regarding the shape of the coastline and its regulation. The high resolution and accuracy of the data obtained make the LiDAR bathymetry technology an excellent tool for mapping, planning, maintaining, and managing national water bodies and coastal regions.

Compared to other measurement techniques carried out from a boat (e.g., single-beam or multi-beam probe), airborne LiDAR laser system bathymeters allow for measurement in non-navigable areas, where so far only direct measurement with devices, such as hand probes and measuring poles, has been possible.

## 2. The Principle of the Green Laser Operation

A laser is a device that emits electromagnetic radiation in the visible, ultraviolet, or infrared range using the phenomenon of forced emission [15]. It consists of three elements, which are: an external pumping system, an excited active medium, and an optical resonator. Usually, the most important feature of lasers is the wavelength of the laser radiation and its power.

**Figure 1:**
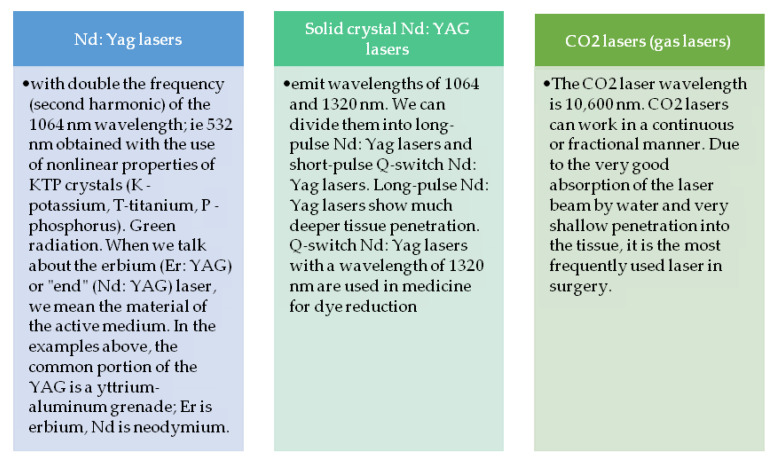
Brief characteristics of the most commonly used lasers.

Due to their power, lasers can be divided into: low power lasers (1 to 6 mW), medium power lasers (6 to 500 mW), and high power lasers (500 mW). The most commonly used lasers include: CO_2_ lasers (gas lasers), solid crystal Nd: YAG lasers, and doubled frequency Nd: YAG lasers (Figure 1).

**Figure 2:**
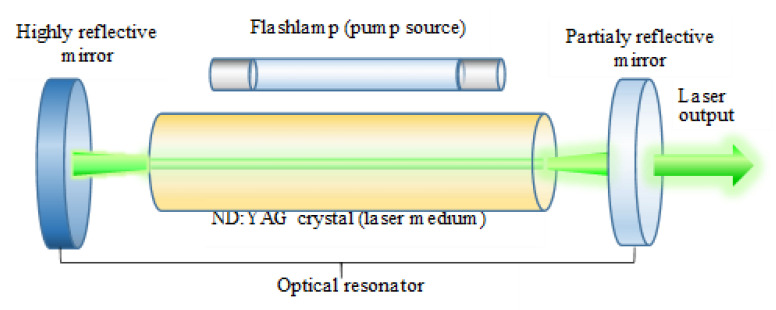
Schematic diagram of a solid-state Nd: YAG laser (green laser).

The energy directed to the active medium by the pump causes the emission of energy in the form of radiation (Figure 2). The excited active medium is located between the mirrors that make up the “optical resonator”. One of these mirrors is a unidirectional mirror. The resonator amplifies the radiation of the excited active medium. However, only some of the radiation can leave the optical resonator through the unidirectional mirror. This radiation in the form of a beam is actually laser radiation. Therefore, in measuring instruments with a green laser beam, it is not a basic laser diode that works, but a complex optoelectric system consisting of a system of lenses, crystals (active media), and filters.

Light particles (photons) excited by electricity emit energy in the form of light.

Laser radiation has four essential properties: coherence, monochromatic, strong beam concentration, and enormous power. Due to these properties, laser light is used in many areas of modern material processing, in medicine or remote surveying.

The laser wavelength determines its allocation to a specific class. The laser classes indicate the safety level of the device emitting laser radiation.

The green laser is the neodymium-yag (Nd: YAG) laser. The light beam is transmitted through the KTP crystal (potassium, titanium, phosphorus). The name green comes from the color of the laser beam which, when passed through the crystal, acquires a specific, strong green color.

The laser is characterized by a wavelength of 532 nm and high energy. The nodymium-yag laser light is actually 1064 nm long, but passing it through the KTP crystal doubles the frequency and shortens the wavelength. This process allows to achieve unique properties that distinguish the green laser from others available on the market.

The green laser, compared to the red one, is characterized by incomparably higher precision, but also requires much more frequent charging of replaceable batteries and their possible replacement. It tolerates low temperatures worse, and in this respect the red ray used in the laser is more durable and more resistant to interference. The differences are very clearly visible on the example of laser levels. The twin CST/Berger ALHVD (red) and CST/Berger ALHVGD (green) lasers have identical batteries, but the first runs for 60 h and the second only runs for 25 h.

The reason for the poor performance at low temperatures lies in the group of crystals (active media) that emit light of the correct wavelength. The group of these crystals is very sensitive to low temperatures. It turns out that, at temperatures below 0 °C, the green beam loses as much as 50% of its visibility. Therefore, green measuring instruments are intended mainly for work in positive ambient temperatures, while the red ones ensure free operation in the range from −20 to 50 °C. Green options are more modest: from 0 to 40 °C.

In geodetic instruments (e.g., rotary levels), the green beam has a power of <5 mW, which means that the laser belongs to the 3R safety class. Looking into the beam through optical instruments can be dangerous, the eyes are protected by instinctive defense reactions (blink reflex). For comparison, the red models meet the safety class 2 or 2M.

Comparing lasers emitting green and red light, it can be stated that the visibility of the displayed beam is different. The human eye is about four times more sensitive to green than to red. Thus, the light of a green laser will be seen by humans four times better than the light of a red laser.

## 3. The Use of a Green Laser in Airborne LiDAR Systems

The green laser can penetrate the water and on this basis, it can provide information about the presence of underwater objects or the bottom topography. The dependence of the penetration of laser rays through water depends on its purity (transparency). This is such a strong and obvious relationship that the penetration depth of a given system is usually given not in the form of the penetration depth expressed in meters of depth, but in the form of a multiple of visibility—the so-called a Secchi disc [16]. The Secchi disc is a device designed by Pietro Angelo Secchi for measuring the transparency of water. The Secchi disc is a white, matte circle-shaped plate with a standardized diameter and white color. It is lowered from a boat in a given water reservoir on a graduated line or a rod with a centimeter scale. The purity of the water is defined by the depth to which the disc is still visible. Penetration of bathymetric systems is in the range of 1–3 depths of the Secchi disc.

The green laser’s ability to penetrate water has been used in LiDAR air bathymetric systems (acronym for “light detection and ranging”).

The most commonly used means for obtaining LiDAR data over large areas are airplanes and helicopters, and more recently, drones [17]. Space platforms are also possible, as in satellite laser altimetry [18]. They use two general types of LiDARs, namely topographic and bathymetric [19]. The former typically uses a near-infrared laser to map the terrain, while the bathymetric LiDAR uses light in the green spectrum as it passes through the water to also measure the depth of the seabed and riverbeds (Table 2).

LiDAR scanners consist of several components. Of course, you need a light source, a laser diode, and its receiver that measures the light reflected from the scanned object. Additionally, they are usually equipped with an optical system that shapes the radiation beam in such a way as to increase the scanning range. LiDAR scanners also include GPS modules that provide location information. In addition, if the measuring device works on board a moving car or aircraft, correction of the results is required, because the calculations must take into account the height at which the vehicle is located and its inclination. Therefore, scanners are equipped with inertial measuring units.

Air LiDARs for topographic mapping usually use YAG diode lasers with a wavelength of 1064 nm, while bathymetric systems (underwater depth research) usually use YAG lasers with a frequency doubling of 532 nm because 532 nm penetrates the water with much less attenuation than 1064 nm.

Some of the existing systems use the unique LiDAR off-camera technology that illuminates objects from multiple angles, minimizing shadowing in the data. LiDAR with off-sky imaging technology is also better for detecting objects on land and in water.

Bathymetric scanners, apart from the fact that they use a green laser, differ from topographic scanners in much greater power, lower frequency of laser pulses, and operation from lower flight altitudes.

The knowledge of the location and orientation of all these elements enables the LiDAR system to record accurate measurements. Some of these sensors can now measure more than 100,000 points per second, resulting in measurements with more than 10 points per m^2^ in shallow water [20]. In a recent study for Samoa, over 1.8 billion points were captured in an area of just over 1100 km^2^. The deepest of these measurements reached a depth of just over 75 m.

### 3.1. LiDAR Bathymetry

Currently, bathymetric measurement platforms include surface ships, underwater platforms, aircraft, and even satellites. In the context of surface navigation, observations are possible from large ships used in offshore research, as well as unmanned, remotely controlled, or autonomous units, preferably used in the study of inland waters (rivers, reservoirs, etc.). When it comes to underwater platforms, autonomous underwater vehicles (AUVs) are commonly used, as well as remotely operated vehicles (ROVs) from a surface vessel, both used for high-resolution deep-water mapping. On these platforms in particular, acoustic sensors are on board, although AUVs and ROVs equipped with light and range sensing (LiDAR) systems and high-resolution cameras are already a reality.

Bathymetric LiDAR is based on the use of blue-green waves that can better penetrate the water. Longer waves cannot be used because they are absorbed by water, and shorter waves would be scattered and absorbed by water particles [21].

The theoretical basis of ALB (aerial laser bathymetry) was developed in the late 1960s [22]. Their implementation was not possible then, due to problems related to the variability of the measured height of the water table. These problems were solved with the improvement of GPS technology in the late 1990s. Technical development made it possible to use aviation LiDAR systems in bathymetric measurements [23].

The concept of the bathymetric scanning system is based on the simultaneous use of two lasers in the light range: infrared and green. The IR pulse reflects off the surface of water or land, while the green pulse penetrates the water and reflects off the bottom of a body of water [24]. The beams are usually not perpendicular to the terrain surface, as in the case of aerial topographic laser scanning, but forward at an angle of 15–20°.

The depth is determined on the basis of the difference in registration time of the beam reflected from the water surface and the beam reflected from the bottom. Thus, the point density significantly depends on the pulse frequency, which determines the type and capabilities of the scanner and the condition of the water during the measurement [25,26]. An alternative method for echo sounder and ALB measurements is the satellite method, which can be used to determine depth especially in large areas of clear waters [27].

**Figure 3:**
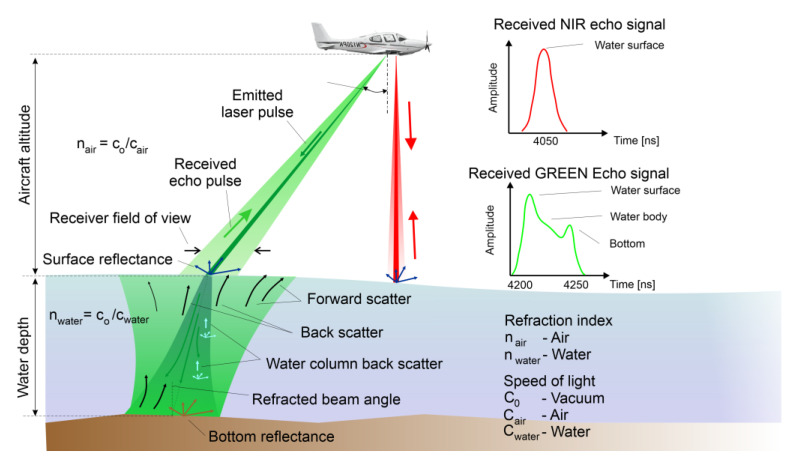
Topographic (NIR) and topo-bathymetric (green) laser scanners mounted on the same airborne platform; (**left**) reflection of NIR signal at water surface; (**right**) principle of airborne laser bathymetry (refraction of laser beam at water surface, echoes from near water surface, water column, and water bottom).

The bathymetric scanner (Figure 3) uses two lasers: a blue-green laser (wavelength 532 nm) and an infrared laser (near infrared, wavelength 1064 nm). The system sends both laser pulses simultaneously. The infrared ray experiences scattering and partial mirror reflection on the water surface, whereas the blue-green ray penetrates the water, is diffused, and partly reflected from the bottom. The water depth is determined based on the difference in distance recorded from both laser beams [13].

When the laser is transmitted in water, the echo power of each part received by the receiver can be regarded as the convolution of the echo signal of each part of the LiDAR and the system response w. The echo power $P{\left(t\right)}_{c}$ of the water body can be expressed as [28]:

(1) $$P_{c}\left(t\right)=\int w\left(t_{c}\right)P\left(z\right)dz\;w\left(t_{c}\right)=\frac{2}{T_{0}}\sqrt{\frac{\ln2}{\pi }}\exp[-4\ln2\frac{{\left(t-t_{c}\right)}^{2}}{T_{0}^{2}}],\;t_{c}=\frac{2H}{v}+\frac{2nz}{v}$$

where $t_{c}$ is the two-way time delay between the detector and the water body, *H* is the working height of the LiDAR, $T_{0}$ is the full width at half height of Gaussian distribution, and $P\left(z\right)$ is the water body LiDAR equation. When O-LiDAR laser propagates underwater, the general LiDAR equation $P\left(z\right)$ of backward elastic scattering of the water body can be expressed as [29,30]:

(2) $$P\left(z\right)=\frac{P_{0}A_{r}\eta O\left(z\right)T{\left(1-l_{s}\right)}^{2}}{{\left(nH+z\right)}^{2}}\frac{v\Delta t}{2n}\left(\beta_{\pi }^{p}\left(z\right)+\beta_{\pi }^{w}\left(z\right)\right)exp(-2\int_{0}^{z}\left(\alpha^{p}\left(z^{\prime }\right)+\alpha^{w}\left(z^{\prime }\right)\right)dz\prime )$$

where $P_{0}$ is the average power of the initially emitted laser pulse, $A_{r}$ is the receiving aperture area, η is the photoelectric conversion efficiency of the detector (determined by the type of material of the photodetector), $O\left(z\right)=\frac{A_{t}}{A_{l}}$ is the overlap coefficient of the detection targets (between 0 and 1, which mainly affects atmospheric signals); $A_{t}$ is the illuminated area of the target, $A_{l}$ is the spot area, n is the refractive index of seawater; H is the height of the aircraft from the sea surface, z is the water depth, $l_{s}$ is the Fresnel Reflection coefficient (the value is usually 0.02 for a perpendicularly incident laser on the sea surface); T is the transmittance of the receiving aperture, v is the propagation speed of light in vacuum, $\beta_{\pi }\left(z\right)=\beta_{\pi }^{p}\left(z\right)+\beta_{\pi }^{w}\left(z\right)$ is the phase function of the 180° scattering angle, $\alpha \left(z^{\prime }\right)=\alpha^{p}\left(z^{\prime }\right)+\alpha^{w}\left(z^{\prime }\right)$ is the effective attenuation coefficient of LiDAR, and the superscripts p and w are expressed as suspended solids and pure water in the water body, respectively.

Acquisition geometry parameters must consider factors such as the angle off nadir at which the pulse is transmitted from the aircraft, or the bathymetric angle of incidence, aircraft altitude, refracted beam angle, and the receiver field of view [21,31,32,33,34,35].

The water depth has a significant effect on the power of the return pulse, as the power decays exponentially with depth [36]. Because depth has such a pronounced effect on intensity values, it is highly important to have accurate depth estimates when calculating the bottom reflectance.

The rate at which the return power decays at increasing depth is described by the diffuse attenuation coefficient. This coefficient is defined by [33,34] as the sum of the absorption coefficient and the backward scattering coefficient. For systems with smaller receiver field of view, it is also important to consider a forward scattering coefficient. 

Compared to the technology of acoustic bathymetry on ships, the LiDAR (ALB) aerial bathymetry technology is characterized by a wide range of operation, high efficiency, and safety.

In relation to the topographic LiDAR, the laser beam in the bathymetric LiDAR has an additional water column to cover. This makes aviation LiDAR bathymetric systems more susceptible to the negative influence of environmental factors than their ground counterparts.

The general procedure for obtaining depth from a LiDAR pulse in air involves measuring the time between the return of the two rebound signals. In practice, since both of these recovery times depend on many environmental and hardware factors, it is necessary to use different correctors to obtain a depth estimate.

Potential false impulses of questionable characteristics, both from environmental and hardware origin, must be removed. Techniques, procedures, and algorithms developed for the SHOALS system are described in [37].

The clarity or lack of water is a major obstacle to shallow water penetration from LiDAR bathymetric sensors. High turbidity, the presence of aquatic vegetation, and the low reflectance at the bottom of the water bodies pose a risk to the success of the test.

The possibility of water penetration by laser light is additionally influenced by the content of substances, such as chlorophyll and suspensions, that absorb specific wavelengths [38].

In the case of very shallow waters, it is difficult to distinguish between the surface echoes and the bottom echo.

In the case of very deep waters, it is not possible to measure their depth using LiDAR aerial bathymetry [3].

These impacts can lead to data gaps, reduced data coverage and the quality of the measurements taken. To minimize them, many factors must be taken into account, such as flying weather, air traffic control, water turbidity, tides, water surface condition, vegetation condition, and ground control availability. Understanding and managing these conditions can mean the difference between success and failure.

#### 3.1.1. Data Processing

Processing of LiDAR data in bathymetry is carried out in the following steps:

- georeferencing raw ALB data,
- noise removal and cloud point classification,
- refraction correction.

The first two steps are used in compiling the ALS data in any case. Refractive correction is a unique activity that is only performed when using laser bathymetry data.

Georeferencing is a multi-stage process that uses data recorded by an integrated GNSS receiver and inertial unit (IMU), position corrections obtained from an external GNSS base station, and on-board equipment calibration data. On the basis of these elements, the precise trajectory of the aircraft flight in relation to the GNSS reference stations is determined, then the observation alignment is carried out in order to determine the position and elements of the external orientation of the scanning system (X, Y, Z, Roll, Pitch, Yaw angles) [39]. These parameters allow to determine the approximate location of the cloud points. Accurate localization is achieved by block alignment of the scan series (strip adjustment). As a result, shifts, drifts, and other systematic errors are corrected between the series and a consistent data set is created. Strips adjustments use common points recorded on adjacent rows and previously measured points or control surfaces. Building roofs are often used as control surfaces. During the alignment of point clouds, the registered planes are compared to determine the shift between field measurements and the cloud. The use of this method in ALB requires flight planning in such a way that at least a part of the series would reflect the terrain, and not only the surface of the water reservoir, as in the case of the work [40]. Surfaces and control points are also used in assessing the accuracy of the results obtained.

Automatic classification of point clouds obtained from ALS is based on the following data: point location, number of the next echo and the intensity of the laser beam reflection, and in the case of full wave form devices, the shape of the echo, the value of the scanning angle, distance from the scanner. These data used in neighborhood analysis, thresholding according to relative height, plane search, reflection intensity and reflection width analysis (full width half minimum; FWHF) allow for qualifying terrain points to classes defined as a widely used standard by the American Society for Photogrammetry and Remote Sensing (ASPRS). One of the basic factors allowing to distinguish water points from other classes is the very low intensity of the laser beam reflection. In the case of ALB, the target classes are defined differently. The basic classification is to distinguish between water surface points, bottom points, and other points (noise, points within the water column, aquatic vegetation, etc.). More detailed classification can be found, inter alia, in studies conducted by the National Oceanic and Atmospheric Administration (NOAA).

The sample cloud point classification made by NOAA [41] includes the following classes:

- 1-Unclassified,
- 2-Ground,
- 7-Noise,
- 25-Water Column,
- 26-Bathymetric Bottom or Submerged Topography,
- 29-Submerged feature,
- 30-Submerged Aquatic Vegetation,
- 31-Temporal Bathymetric Bottom.

**Figure 4:**
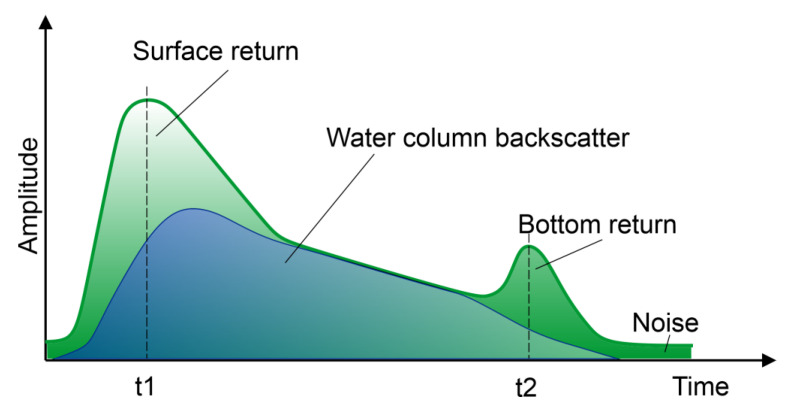
Schematic green LiDAR waveform showing the three principal signal components.

In the case of ALB classification, the echo analysis is extremely important, especially given that the registered intensities of laser beam reflection are much lower than in the case of objects on the earth’s surface. Schematically, the response curve of the ALB signal is a combination of the three main components (surface return, water column backscatter, bottom return) (Figure 4).

In fact, the shape of the response curve can be much more complicated due to the presence of suspended solids and living organisms (including phytoplankton) in the water. In addition, in shallow tanks, the surface and tank bottom response peaks may overlap. These factors make proper analysis a big challenge. Currently, there are three groups of LiDAR full waveform analysis methods [42]:

- Deconvolution methods: such methods are used to remove the transmitted waveform component from the received signal to obtain the surface or bottom response [43,44].
- Echo detection: this is a group of methods that does not take into account the radiometric features of targets, but locates echoes by a direct indicator, e.g., a threshold, center of gravity, zero crossing of the second derivatives [45].
- Mathematical approximation: consisting in fitting mathematical functions to the LiDAR waveform with parameters that allow to determine the position of the targets. Gaussian function sets, lognormal function, or Weibull function [46] are widely used.

As indicated by Ding et al. [47], the most commonly used is the triangular fitting algorithm (TF), which uses the Gaussian function to approximate surface and bottom components and the triangular function to fit the water column component.

To meet the seafloor topographic accuracy demand of the International Hydrographic Organization (IHO) [48] Standards for Hydrographic Surveys, refractive correction is required. As indicated by Su et al. [49], the greatest source of uncertainty in ALB measurements is the laser pointing uncertainty and refraction uncertainty on the sea surface.

In the correction of refraction, at least two physical phenomena are taken into account: refraction of the radiation beam passing through the water surface and lower speed of the laser beam propagation in the water medium.

To correct refraction, it is necessary to create a water surface model. This model allows for the determination of the local angle of incidence of the laser beam on the water surface and the time of the radiation course in the water medium. This model is created on the basis of classified cloud points from the green scanner or points recorded with the NIR scanner. In the case of the green scanner, the effect described by (Mandbulger G. et al., 2013) [13] should be taken into account, which consists in the fact that the points recorded by the green scanner on the water surface are approximately 10–25 cm below the corresponding points from NIR scanner. The density of the points used to create the water surface model is of fundamental importance here. In the case of a sparse mesh, local fluctuations in the water table due to, e.g., undulations, may be of great importance for the accuracy of the correction. The existing methods of wave refraction correction use various simulation methods using the wave spectrum (Birkebak et al., 2018) [50], (Dong et al., 2020) [51] or summing series of periodic functions (Westfeld et al., 2017) [52].

The actual angle of incidence of a laser pulse entering water (α) is the angle between the normal of the water surface and the direction of the incident beam. Knowing the refractive indexes of air (n1 approximately equal to 1.0) and water (n2 approximately equal to 1.33), it is possible to determine the refractive angle of a wave of a specific length on the water surface. The refractive index can be accurately calculated based on water temperature, salinity, and depth. The current angle of refraction on the water surface can be determined using Snell’s law [53].

(3) $$\beta =\arcsin\left(\frac{n_{1}\sin\alpha }{n_{2}}\right),$$

On the basis of the same law, it is possible to determine the speed of electromagnetic wave propagation in the water medium, which in turn will allow to determine the correction to the distance recorded by the laser scanner. Both of these values allow for the correction of the position of each point of the cloud.

#### 3.1.2. Review of Bathymetric Scanners

The first measurement system allowing for the simultaneous measurement of the topography of the area and the depth of a water reservoir was introduced to the market in 2001 [54]. Since then, new sensors have been developed to efficiently measure both topography and to perform shallow water bathymetric measurements from an airplane or drone.

LiDAR bathymetric sensors have more individual characteristics and differences than LiDAR topographic sensors [55,56]. Importantly, all modern LiDAR bathymetric sensors, apart from bathymetry, can measure topography. The most obvious division is between shallow water (<10 m) and deep water (>10 m) systems. Platelet systems typically have lower laser power per pulse, higher measurement frequency (high resolution), smaller laser trace diameter, and receiver field of view smaller, and can generally only measure water depth in the visible water column. LiDAR deep water bathymetric systems use higher laser power per pulse, lower measurement frequency (low resolution), larger laser footprint and receiver field of view. These deep water LiDAR bathymetric systems differ in their penetration depths from 2.0 to 3.0 times the Secchi depth measurement.

The water depth has a significant effect on the power of the return pulse, as the power decays exponentially with depth [21,36]. Because depth has such a pronounced effect on intensity values, it is highly important to have accurate depth estimates when calculating the bottom reflectance. The rate at which the return power decays at increasing depth is described by the diffuse attenuation coefficient. This coefficient is defined by [33,34] as the sum of the absorption coefficient and the backward scattering coefficient. For systems with smaller receiver field of view, it is also important to consider a forward scattering coefficient [31,32].

Scanning patterns for sensors consist of shape, slope, and method. The scan shapes vary between straight, elliptical, circular, elliptical, and circular arcs. Circular and elliptical scanners can look forward and backward, increasing the area sampling number, although this may result in oversampling along the edge of the scan. The other shapes are usually tilted forward or backward with respect to the plane. Scanning methods differ between rotating or non-rotating, using different optical deflecting elements, such as prisms, gratings, and mirrors creating specific scan patterns. All of these methods result in subtle differences in the scanning pattern. The first mechanism, the oscillating mirror, is based on pendulum motion, which results in a scanning trace similar to the shape of a sinusoid. The resulting point cloud density varies depending on the distance from the center of the scan lane, which is the result of the mirror’s inconsistent velocity when changing its direction of movement. Another mechanism, the rotating polygon, is a system that results in an even distribution of points on the surface of the earth. The mirror consists of several sides constituting reflecting surfaces and rotates in a uniform rotational motion. The Palmer scanner is a mirror whose movement causes the laser beam to be constantly deflected by a given angle. The result is an elliptical scan trace. The Palmer scanner is popular in bathymetric scanners due to the ability of the beam to pass through the water surface and obtain measurements of the bottom of the tank thanks to the constant angle of incidence of the laser beam on the surface of the water. The fourth distinguished mechanism is a fiber optic system, in which glass fibers are used to determine the direction of incidence of the laser beam, illuminated by a laser beam by a rotating mirror. This solution is characterized, on the one hand, by high stability of the generated point distribution. On the other hand, it results in low flexibility of the mechanism. An important consideration when using LiDAR bathymetric systems is the laser energy per pulse. Although factors, such as the receiver telescope surface and field of view, affect penetration depth, laser power combined with pulse duration has the greatest effect on depth penetration. High laser power and pulse duration lead to deeper penetration of the water column. The disadvantage of the higher laser energy per pulse is that the measurement frequency is lower, resulting in a lower point density. The selected, currently available sensors [57,58,59,60] measuring from the aircraft ceiling and their characteristics are summarized in Table 3.

The penetration depth of the water column depends on the laser power, which is correlated with the pulse duration. The higher the laser power, the deeper the penetration, but at the same time the lower the measurement frequency. The main factor behind the use of limited laser energy is eye safety standards.

The decision on the choice of a particular system should depend on the area of the proposed research, the nature of the research and its purpose. The most common aspects that influence the choice of a LiDAR bathymetric system concern the maximum and minimum measuring depth that must be achieved, the level of detail of the measurements performed, and the products that are to be the end result of ALB measurements (profile/DTM).

Bathymetric measurements using a green laser are also performed from the UAV [61]. Currently available drones equipped with a bathymetric LiDAR allow the measurement of up to 40,000 points per second on a deep bathymetric channel and up to 140,000 points per second on a shallow bathymetric channel. At the same time, the measurement carried out on the topographic channel allows the measurement of up to 500,000 points per second. This allows you to collect data with the required detail and resolution, characteristic of topographic LiDAR applications. In terms of coastal mapping and water measurements, this can be done down to a depth of 25 m. The acquisition of data with high accuracy and density on the bathymetric channel is constantly being improved and developed (some manufacturers confirm the possibility of making measurements up to a depth of 50 m).

## 4. Use of LiDAR Bathymetry

Aerial laser bathymetry (ALB) is an attractive technology for measuring shallow waters due to the speed of data acquisition and high point density achieved. Especially valuable is the possibility of using ALB in non-navigable areas, where an alternative is traditional, ground-based geodetic surveys that require entering the water by surveying (wading with a pole). Compared to underwater acoustic systems, ALB is suitable for large areas, providing dense and accurate data [62].

Most of the scientific literature published so far has focused on the use of ALS in coastal areas [63], while similar studies in river environments are considered less frequently [64,65].

### 4.1. Application for Measuring River Crosses and Fluvial Processes

Flowing water is one of the basic factors that shape the Earth’s surface. River processes are defined as the physical interaction between flowing water and the natural channel through which it flows. River processes can be divided into [66]:

- river erosion, i.e., cutting into the Earth’s surface, we distinguish erosion: deep, backward and lateral,
- transport or transport of rock material downstream of the river,
- accumulation, that is, the deposition of material carried by the river.

A number of modern measurement techniques are used in the study of river environments. The instruments used allow for quick data acquisition, but measurements taken from the ground surface [67,68,69] are limited to selected areas of smaller rivers. The density of the riverbed points obtained by classical methods is low and heterogeneous compared to the possibilities of the ALS.

Topographic LiDAR can be used to capture the coastal area [70,71,72], but the used IR beam is absorbed by water and does not allow the bottom to be measured. Measurement of the water depth is also possible on the basis of RGB and/or hyperspectral images. It is made on the basis of the correlation between the depth of water and the color of the image [73,74] and is often combined with LiDAR and/or ground data [75,76,77,78].

Most of the studies mentioned on the evaluation of the deposited sediments and the size of erosion are performed using the DEM of differences (DoD) models [79] as:

(4) $$z_{\mathrm{DoD}}=z_{\mathrm{new}}-z_{\mathrm{old}}$$

where $z_{\mathrm{DoD}}$ is a single DoD cell deposition/ erosion value and $z_{\mathrm{new}}$, $z_{\mathrm{old}}$ corresponds to the height of the DTM cells at the epochs studied.

Based on the estimation of the DTM errors that propagate in the DoD, it can be assessed whether the difference is a measurement noise or represents a real change:

(5) $$\sigma_{DoD}=\sqrt{\sigma_{DTM,\;new}^{2}+\sigma_{DTM,\;old}^{2}}$$

where $\sigma_{DTM,\;new}$, $\sigma_{DTM,\;old}$ represent the spatial accuracy of the DTM.

Aerial LiDAR bathymetry (ALB) has evolved rapidly in recent years and now enables high resolution (>20 dots/m^2^) and height accuracy (<10 cm) mapping of river topography for both water and coastal areas [80].

The spatial resolution of the ALS data is suitable for the visual recognition of macroscale forms of river land such as river channels, palaeochannels, alluvial fans, levees, and valley edges [72,81,82,83]. One-dimensional river basin profiles can be used to distinguish the morphological units of the riverbed [84]. A distinct advantage of LiDAR data is that it allows for faster, more accurate, and detailed mapping of river landforms compared to lower resolution elevation data obtained from surveys. off-road.

The vertical error and spatial resolution of the data define the minimum size of the form that can be identified [72]. The paper [80] proposed a method for determining air/water-interface when the echo density of signals reflected from the water surface is low (the echo coverage of the water surface was only 25%, counting cells of 1 m^2^ with at least a single surface echo as important). The method is based on generating a model of the riverbed from rarely captured cross-sections.

**Figure 5:**
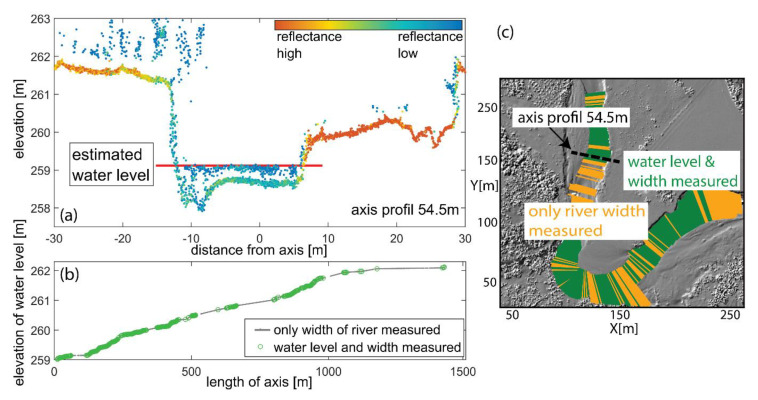
Semi-automatic derivation of water surface model; (**a**) cross section with LiDAR echoes colored by reflectance and manually defined water level and extent (red line); (**b**) longitudinal section with measured water levels (green circles) and/or extents (gray dots); (**c**) plan view, measured sections marked in green/orange [80].

The input in this method is the 3D LiDAR point cloud and the 2D river axis. On their basis, transverse sections perpendicular to the river axis are generated. Assuming a constant water level in a given cross-section, the observer, in a manual way, in the graphical editor defines the height of the water level in each subsequent cross-section. In the next step, the height of the water table is extrapolated in subsequent sections and subsequent sections are shown on the terrain plan and longitudinal section (Figure 5)

**Figure 6:**
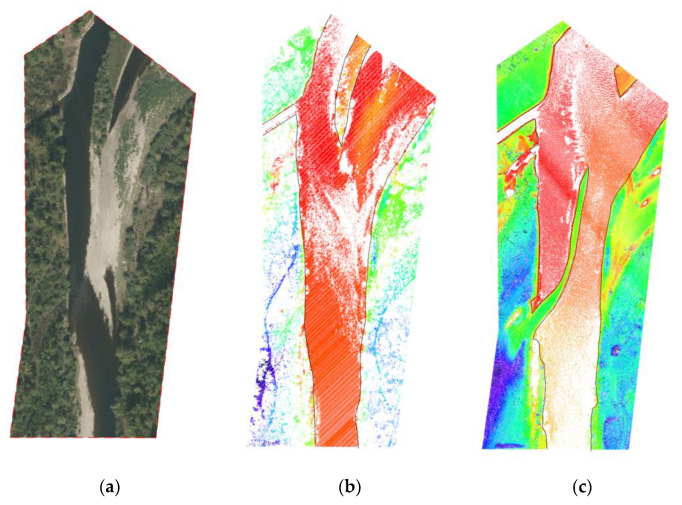
Change of the river bed in the period 2012–2020 (Soła River, Poland) (**a**) orthophoto, (**b**) LiDAR 2020, (**c**) LiDAR 2012.

Sedimentation processes caused by the transported material influence channel forming flows (Figure 6) [85]. On the other hand, serially repeated topographic measurements allow for research on changes taking place after periods of high water levels [67].

For the needs of the national authorities, assuming different variant data, hydraulic models are made, using computer simulation to assess the risk of flooding. Planning work carried out in this way is based on the scientific, evidence-based basis of the flood risk assessment that is required as part of the planning process. Hydraulic models use long-term statistics on precipitation, sea levels and river flows, along with detailed simulations of how water in the landscape is moving. These activities are aimed at determining the likelihood of a flood occurring in an independent and man-dependent manner (e.g., by the proposed investments).

As a consequence of the high resolution of LiDAR data, it is possible to use them to improve the performance of the 1D [86,87] and 2D hydraulic models [88,89], as well as to circle the height of the water table [80].

For one-dimensional (1D) hydraulic surface water models, single water surface ordinates are computed at each section where the flow is shown only perpendicular to the section and must be drawn by the model builder. In a 2D model, the digital elevation model (DEM) serves as the basis for calculations to determine the depth, velocity, and direction of surface waters.

**Figure 7:**
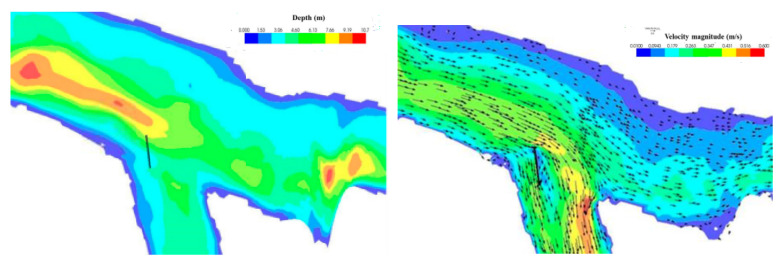
Simulated flow depth and velocity distribution with its vectors (modified after [90]).

With one-dimensional (1D) surface water hydraulic models, single water surface elevations are computed at each cross-section where the flow is only shown perpendicular to the cross-section and needs to be drawn by the model builder. In a 2D model, a digital elevation model (DEM) is used as the basis for computations to determine surface water depths, velocities, and directions (Figure 7).

**Figure 8:**
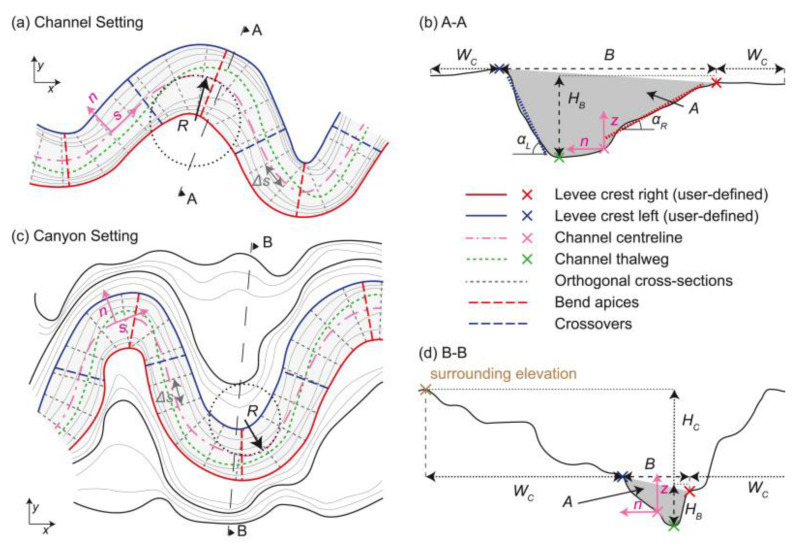
Determination of morphometric parameters of the river bed [91]. User-defined levee or bank crests (right in red, left in blue) for bankfull active channel in a channel (**a**,**b**) and canyon setting (**c**,**d**) and user-defined corridor width (*W_C_*) outside bankfull active channel.

Article [91] presents a new Matlab script for determining the morphometric characteristics of rivers, canals and canyons. On the basis of the trough edges, previously defined by the user, the script determines the center line and other morphometric features, such as the edge width (B), the radius of the centerline of curvature (R), and waviness (SI) and determines the orientation of the sections, the location of the vertices of bends and intersections. The script is also resistant to very sharp turns and irregular troughs with sudden changes in curvature. If bathymetric or digital elevation topographic data (derived from Bathymetric LiDAR) is available, the script provides additional morphometric features, such as thalweg, slope (S), slope depth (HB), section area (A), channel aspect ratio (B/HB), and shaft inclination (α) (Figure 8).

Steep riverbanks can be difficult to measure altitude with ALS. This was demonstrated in the work of Hodgson and Bresnahan [92]. This is in line with the finding of Hyypp et al. [93], who observed that the DTM altitude error determined from the LiDAR observation significantly increased on slopes covered with trees with a slope greater than 15 degrees. Another problem may be the fact that the point density obtained from ALS may be too sparse, resulting in riverbanks not being accurately described in the terrain model.

### 4.2. Application to Measurement of Shallow Offshore Sea Zones and Abrasion

As sea levels rise and the severity of extreme natural phenomena increases, the need to deepen the knowledge of the coastal zone becomes more and more evident. The basis for understanding the risk in areas exposed to the negative effects of sea waters is the determination of the course of the coastline and the land and sea coastal surface features.

The intensity of the sculpting activity of the sea depends on the type of rocks that make up the shore, the local relief, sea tides and the location of the body of water. The abrasion consisting in the gradual eroding of the seashore especially concerns the cliffs. Directly below the eroded high coast, an abrasive niche is created, and the eroded, fragmented material is transported and accumulated.

Accumulation occurs most often on the low coast, where the accumulation exceeds erosion and gives rise to forms such as beach, shoreline, storm embankment, revue, lido, lagoon, etc. Bathymetric LiDAR is the most effective and cost-effective technology that allows simultaneous recording of land and sea bottom for obtaining a continuous, detailed 3D elevation model along the coastline [94]. Its ability to successfully capture heights on both sides of the coastline, in areas extending more than 100 km along the coast, made the bathymetric LiDAR the “gold standard” for coastal sensitivity and coastal benthic habitat modeling.

Bathymetric LiDAR can be successfully used to create high-resolution bathymetric maps, which represent the basic data set for understanding the impacts and threats of climate change, erosion trends, and sea level rise [95,96,97,98].

In addition, the time series of ALB datasets can successfully support accurate change detection analysis in this difficult environment [99].

**Figure 9:**
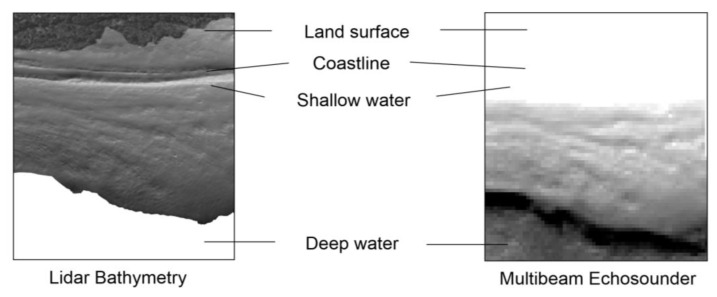
Comparison between bathymetric LiDAR and multibeam echosounders (Dobczyce reservoir, Poland).

The use of LiDAR airborne bathymetry (ALB) has become a common technology for mapping shallow areas in high resolution [100]. Compared to underwater acoustic systems, ALB is suitable for large areas, providing dense and accurate data (Figure 9).

Another problem that is important in coastal protection and management is the definition of the coastline. Data from measurements at a depth of 0–2 m are usually incomplete or not available at all due to the difficulty of reaching such shallow waters [101]. LiDAR Bathymetry (ALB) is an active remote sensing instrument used to obtain the topography of such shallow coastal waters [102]. It can efficiently deliver high accuracy and density bathymetric data sets in non-navigation and complex topographic areas [103].

**Figure 10:**
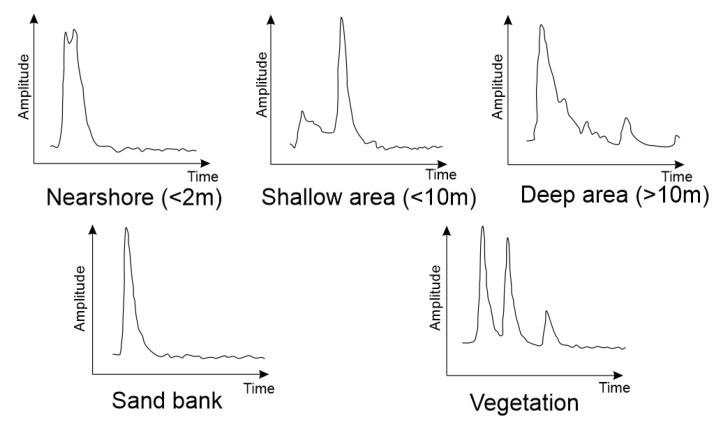
Echo responses for bottom topography elements (based on [105]).

Due to its excellent spatial positioning performance, ALB is widely used for the seamless topobatimetric mapping of shallow water areas, such as reefs near islands [104] (Figure 10).

ALS data can record the relief of the seabed and adjacent land surface. The numerical terrain model created on their basis allows for a precise determination of the state of the coastal zone at the time of registration. The performed cyclical measurements allow for the monitoring of the coastal zone, identification of trends in changes in the shape of the coastline, designation of relatively safe places, not endangered by erosion and flooding, and particularly endangered places. In the field of underwater measurements, attention should be paid to such possibilities of LiDAR bathymetry, such as identification of types of substrate forms and anthropogenic structures, automatic mapping of seabed geomorphology [40], identification of erosion and deposition patterns along coast [106], assessment of the rate of accumulation of post-flotation sediments at the bottom of the reservoir [107], and underwater landslide research [108].

The airborne LiDAR (ALB) bathymetric system is widely used in describing the topographic features of the seabed, building 3D models of the seabed, monitoring coral reefs, and underwater archeology.

With regard to the classification of the seabed relief, the bathymetric LiDAR has not been widely used so far. Applications potentially available in the literature include shallow coastal area monitoring [109,110], monitoring the status of navigation and protection channels of the structure [111], classification of the tidal environment [112], benthic habitat mapping [36], as well as the organization and placement of archaeological sites in shallow waters [113].

Moreover, from ALB, sedimentological (density, concentration) and hydrodynamic (suspended sediment concentration, turbulence) information can be obtained on the basis of the analysis of laser return intensity curves [114].

**Figure 11:**
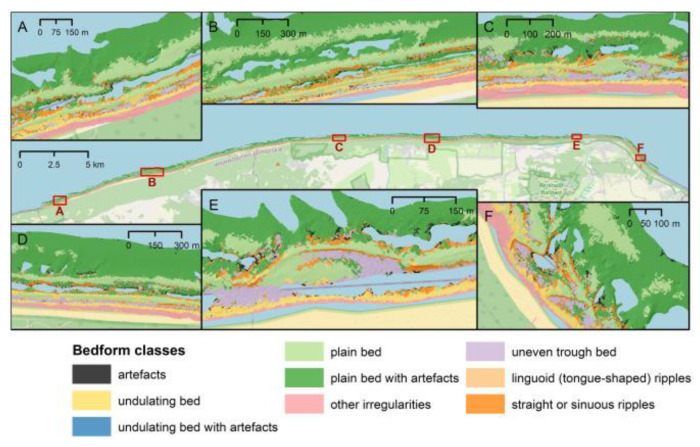
Classes of geomorphological bedforms automatically mapped using ALB and machine learning supervised, (**A**–**F**) subsets of the study site marked on the general map [40].

In the study [40] concerning the Polish coast of the southern Baltic Sea, exposed to increased coastal erosion, the recognition and classification of geomorphological forms occurring in the ring in both natural and anthropogenic sections of the coastal zone was made (Figure 11). Machine learning classification results were compared with the manual characteristics of seabed forms and coastal protection structures, and machine learning classification procedures for ALB were assessed.

Identification and classification of seabed geomorphology based on remote sensing data (ALB) can be done manually (preferably by one interpreter [115] or by automatic or semi-automatic. Seabed classification methods include unsupervised or supervised approaches. In the first approach, seabed classification is based on properties and relationships, e.g., under Jenks’ unsupervised classification [116]. The second approach involves training with an input data set that can be defined manually or in the field [117]. Both techniques can be applied by performing image pixel analysis or based on image analysis based on geographic features. The latter method has been used in seabed research for over 20 years [118].

Testing the waters with green LiDAR light allows for precise mapping of the bottom of the reservoir in terms of the presence of undesirable objects (explosives, wrecks, garbage, etc.). The possibility of searching for crude oil and natural gas deposits using the DIAL technique is also noteworthy.

The results of research conducted on the basis of ALS data can be used:

- by the state administration responsible for the safety of the seashore in order to select appropriate methods of its protection against erosion;
- with safe planning of investments in the coastal zone and preparation of sea space development plans;
- by local self-government authorities when verifying spatial development plans of seaside towns and making prudent decisions as part of integrated coastal zone management.

## 5. Directions of Bathymetric LiDAR Development

LiDAR is a tool for civil (commercial), administrative, and military use. There is a growing number of industries in virtually every branch of the economy and research activity. The LiDAR technique can be used to create digital three-dimensional representations of areas on the Earth’s surface and the bottom of water bodies by terrestrial, satellite, aerial and mobile techniques. It is widely used to create high-resolution maps and is used in surveying, geomatics, archeology, geography, geology, geomorphology, seismology, forestry, atmospheric physics, laser guidance, aeronautical laser mapping (ALSM), and laser altimetry. LiDAR is currently the most detailed and accurate method for creating digital terrain models.

ALB aerial bathymetric scanning is a promising technique for measuring the bottom of water reservoirs. This technique has developed a lot in recent years due to the capabilities of the scanner as well as the capabilities of post-processing software. Bathymetric LiDAR is a technology for acquiring data from the air. Unlike the topographic LiDAR in air, which uses 1064 nm infrared, bathymetric LiDAR systems use a 532 nm wavelength to penetrate the water column to measure the bottom of a body of water.

Bathymetric LiDAR is currently the most effective and cost-effective technology to simultaneously record both the terrain surface and the bottom of water bodies to obtain a continuous, detailed 3D model of the measured terrain.

When selecting and using a LiDAR bathymetric sensor, environmental factors and individual features of the system should be taken into account. Even then, the operator’s knowledge and experience often determine the success of a measurement. In addition, the decision to select the best test system for testing will depend on the test area, environment, design requirements, and sensor availability. The considerations that typically determine sensor selection are maximum depth, point density, coverage, end product requirements, and, not least, the intended purpose of the data.

Recent advances in LiDAR bathymetric sensors are going in many different directions. Some of these achievements include efficiency gains by increasing point density and penetration depth while maintaining equal accuracy over clear and cloudy water [119], and fast and automatic work with LiDAR data allowing for automatic calibration, registration and refraction correction, full wave processing, quality control, and data export. A solution to the problem of distinguishing signals reflected from the water surface and the bottom under shallow water conditions (less than 2 m) is also being developed [120]. Moreover, this is only the beginning of the use of LiDAR bathymetric sensors in small unmanned aerial vehicles (UAVs), although this is likely to change over the next decade. Cloud computing and big data processing are also very promising, and it will be fascinating to see how the industry takes advantage of these advances to provide additional end-user opportunities.

The LiDAR technique has many significant advantages that distinguish it from other measurement techniques.

The laser scanner is independent of lighting conditions (it is an active system). Nighttime imaging is even more advantageous due to less air turbulence.

Imaging with this technique is possible even when it is completely cloudy (only conditions limiting the penetration of the laser beam, e.g., heavy rain and fog, represent an obstacle). Spatial data are recorded directly, with a very high accuracy that characterizes elevation data. It is possible to register many reflections (signal echoes), and the cycle of data processing and production of final products is short.

Limitations of LiDAR include lower accuracy relative to altitude accuracy, large volume of data, and relatively high cost of data acquisition and processing.

As LIDAR systems evolve, it becomes more and more useful in many applications. In the future, the entire world will basically depend on various LIDAR systems to detect, collect and update data. So far, the greatest progress has been made in obtaining high-accuracy data by developing their correction and calibration. Research will continue to be undertaken to assess the impact of the laser wavelength on the accuracy of the measurement process (the reflection coefficient can only occur at a certain wavelength). Another direction of the conducted research is to draw attention to the scanning method that can be applied using various mechanisms.

The proliferation of unmanned aerial vehicles (UAVs) will force the use of light and small LiDAR sensors, so work will continue on their continuous miniaturization while maintaining the measurement accuracy parameters. Work will be continued on increasing the density of measurement points in the aquatic environment for better detection of details, objects, and identification of the water table.

The automation of data processing processes will constantly progress to increase efficiency and deliver results faster.

Certainly, in some areas, the collected data will be made available on online platforms, which will force improvements to cloud services.

If we care about a high-quality digital surface model (DSM), a point cloud consisting of points that are the first reflection requires manual editing. Fully automatic filtration methods for this type of points for DSM construction are still under development.

The development of laser scanning systems is aimed at increasing their efficiency and increasing the number of registered reflections (echoes) of a single laser pulse. The increase in efficiency is achieved by increasing the frequency of generating laser pulses. In the field of data processing, the methods of aligning blocks of airborne scanning series as well as methods of point cloud classification and filtration are being improved. Given the huge datasets, automatic methods apply here.

The use of data from airborne bathymetric scanning allows the detection of objects located on the seabed. However, post-processing related to point cloud classification is required beforehand, which leads to the separation of classes that guarantee the correct identification of objects, which affects security and indirectly protects the natural environment. Unfortunately, the transparency of water and the density of the point cloud significantly affect the size and efficiency of detecting objects lying on the seabed. Acquisition of data by aerial bathymetry is cheaper and faster. Therefore, it seems reasonable to search for methods of automatic detection of underwater objects, which will be a further stage of research and will be based on the use of full waveform parameters of aerial bathymetric scanning and neural networks.

One of the directions of ALB development is the use of machine learning methods. The authors examine the possibilities of using many algorithms from the machine learning family, such as random forest (RF), support vector machine (SVM), or deep learning, etc. One of the directions of using machine learning methods in the analysis of scanning data is noise removal. Hu et al. [121] proposes a method based on convolutional autoencoding neural networks (CAENN) for denoising the ALS return signal. According to the authors, this algorithm has a strong adaptive ability, and its excellent denoising effect in relation to the methods used so far, although it has not yet been studied well. Machine learning methods in ALB are further developed in point classification. Kogut and Weistock [122] compare support vector machines (SVM) with the random forest (RF) algorithm for classifying water table and bottom points. Very high classification accuracy of both methods applies to points of the water surface and seabed reaching 100%, although for the remaining objects it was only 60%. Similar values were recorded by Kogut and Slowik [123] who used multilayer perception (MLP) artificial neural networks (ANN) and comparatively SVM, random forest, and RUSBusted trees to classify sea surface and seabed points and to detect the location of artificial objects on the seabed.

Due to the fact that the original lidar echo carries a lot of information, the use of machine learning may, compared to traditional methods of classification, offer greater possibilities, e.g., for mapping the formations that build the bottom or aquatic vegetation habitats.

This type of research on aquatic plant habitats is presented by [124,125] in which machine learning techniques are used (Random Forest). The authors use single wavelength or bispectral barymetric lidar. The results obtained have a classification accuracy of up to 85%, which, as the authors themselves indicate, can be improved by using various methods of filtering points with a low prediction confidence. Similar studies on the use of supervised classifiers in mapping seabed morphology are presented by Janowski et al. [40].

Deep learnin-based classification of scanning data is one of the main fields currently being developed in photogrammetry and remote sensing [126,127]. This type of research is also conducted in lidar bathymetry. Shanjiang et al. [128] used the original bispectral bathymetric echo to classify points (sea-land). For this purpose, a multi-layer fully connected neural network and a one-dimensional convolutional neural network (1D CNN) were utilized. They obtained classification accuracy of up to 99.6%, although these are the best quoted data out of 200 attempts. A key factor in obtaining high-quality results is the appropriate number of network training epochs. Mismatch of the training series may result in under- or over-fitting of the network. In the works [129,130] summarizing the current applications of deep learning in laser scanning, further dynamic development of these techniques in the classification and detection of objects is predicted.

## 6. Summary

Measurement of the bottom of water reservoirs and the surrounding area is possible with the use of a number of different measurement systems and calculation tools. So far, the availability of water areas for remote measurements has not been uniform. Unnavigable areas were completely deprived of the possibility of remote determination of the depth and shape of the bottom. Green lidar bathymetry has filled this gap.

The article discusses in detail the basics of the green laser operation and compares its characteristics with the description of the other, most commonly used lasers. The basic feature of the green laser is the ability to penetrate water and it was this feature that encouraged use next to sound waves (used in echo sounders) to measure the bottom of water reservoirs.

Water penetration by the laser beam, however, is limited by water transparency. Therefore, this technology can be used in shallow water areas, and the measurement reaches a depth of 3 Secchi.

The laser can be used from a boat, plane, or drone. The use of LiDAR airborne bathymeters is more advantageous as it opens the possibility of conducting research in areas previously inaccessible for remote measurements.

The parallel use of two wavelengths 532 nm and 1064 nm (bathymetric scanner and topographic scanner) provides a set of data in a very short time and without the need to access the tested object. The IR pulse reflects off the surface of water or land, while the green pulse penetrates the water and reflects from the bottom. The results of these measurements make it possible to measure the height of the water table, measure the shape of the bottom of the water reservoir and its surroundings, and determine the course of the shoreline.

Data processing from bathymetric LiDAR measurement consists of georeferencing raw data, noise removal, point classification and refraction correction. The measurement results can be used to assess river erosion, the rate and size of the transported material at the place of its accumulation. They allow for the identification of forms occurring at the bottom of the channel, for the preparation of cross-sections and longitudinal sections, taking into account the height of the water table, which gives the basis for determining the slope, as the height data is subject to a very small error (they are accurate).

The consequence of having high-resolution bathymetric LiDAR data is the possibility of using them to improve 1D and 2D hydraulic models, determining depth, flow velocity, and direction of flow. As a consequence, this gives the opportunity to design a stream restoration design.

The morphometric parameters of the river channel can be determined on the basis of bathymetric LiDAR data in a precise and reliable way. They have a small error and are based on a large amount of measurement data.

The use of bathymetric LiDAR in marine, shallow coastal zones allows for the assessment of the degree of abrasion and accumulation, as well as for the identification of underwater forms of the bottom relief, sunken wrecks, or underwater archaeology.

The directions of development of LiDAR bathymetry are aimed at not only hardware improvement, but also computational improvement (processing of measurement data). New applications for Lidar bathymetric sensors are still being found. The results of the conducted research are not only of scientific importance, but can also be used by the administration for the purposes of planning, erosion prevention, or ensuring safety.

## Figures and Tables

**Table 1:** Technical comparision of sounding bathymetry methods.

	SBES-Single Beam Echo Sounder	MBES-Multi Beam Echo Sounder	Green Laser Bathymetry
System location	boat	boat	boat, plane, drone
Working area	Navigable water area	Nawigable water area	Shallow water area (up to 3 i Secchi depth)
Measurement coverage	Only a profile along a boat’s route	100%	100%
Type of wave	Sound wave	Sound wave	Green wavelength of 532 nm
System components	Echo sounder, positioning system (GPS-RTK)	Multi-beam head integrated with a motion sensor and a sensor for measuring near-surface speed of sound in water, positioning system (GPS-RTK)	GPS receiver, inertial measurement unit, laser scanner, signal receiving sensor
Frequency	Two frequencies at once e.g., 38 kHz/200 kHz	Between 100 and 700 kHz	1.5 kHz to 512 kHz
Characteristics of the measurement results	Low data density, small measurement errors	Huge data density, all disturbance and noise that need to be eliminated during data processing are recorded	Huge data density, the frequency of their acquisition and the speed of acquisition
Measurement result	echogram	Point cloud	Point cloud

**Table 2:** Comparison of typical parameters of topographic and bathymetric scanners.

Parameter	Topographic Scanner	Bathymetric Scanner
laser wavelength	1064 nm (IR)	532 nm (green)
sent pulse beam divergence	narrow (0.3 mrd)	narrow (0.3 mrd)
return pulse beam divergence	narrow (0.3 m from a height of 1000 m)	wide (2 m from a height of 300 m)
frequency of pulse generating	big (up to 400 kHz)	small (1–10 kHz)
pulse width	short (5–10 ns)	short (<5 ns)
energy emitted	small (5–10 μJ)	big (5–10 mJ)
incidence angle	nadir (0°)	forward (15–20°)
laser sensor	single laser	double (2 wavelengths)
accuracy of distance measurement	1–3 cm	3–5 cm
Scan trace	Parallel lines, sinusoidal	Elliptical lines (Palmer scanner)
Optical sensors	MS digital camera	HSI/MS digital camera
georeference	GNSS/INS	GNSS/INS
platform	helicopter plane	airplane, helicopter, drone
flight altitude	500–1000 m (and more)	300–500 m
processing	Discrete reflections, full wave shape	Full wave shape

**Table 3:** Specification of selected ALB sensors taking measurements from the plane.

	Optech CZMIL Supernova	USGS EAARL-B	Fugro LADS Mk-3	Riegl VQ-820-G
Typical Sensor Environment	Topo-Bathy	Topo-Bathy	Bathy	Topo-Bathy
Laser Wavelengths	Green 532 nm Infra-Red 1064 nm	Green 532 nm	Green 532 nm	Green 532 nm
Scan Shape	Circular	Elliptic Arc	Rectilinear	Elliptic Arc
Scan direction and Angle from Nadir	Fwd and Aft 20°	Fwd 5° Sideways 22°	Fwd up to 8°	Fwd or Aft 20°
Scan Method	Rotating Prisms	Oscilating Raster Scanner	Oscilating Mirror	Rotating Multi-Facet Mirror
Lase Energy Per Pulse (Green 532 nm)	3 mJ	0.4 mJ 0.13 mJ per beam	7 mJ	0.02 mJ
Pulse Duration	2.0–2.2 ns	0.85 ns	6.5 ns	1.2 ns
Peak Measurement Frequency	10 kHz@532 70 kHz@1064	15 kHz or 30 kHz	1.5 kHz@532	Up to 512 kHz@532
532 nm Nominal Footpront Diameter Water Surface (1/e^2^)	2.4 m	0.3 m per beamlet 1.6 m apart	3 m	0.6 m@AGL Below
Nominal Flying Height	400–800 m AGL	Nominal 300 mAGL	400–915 m AGL	Nominal 600 m AGL
Swath Width (as a function of point spacing or altitude)	291 m@400 m AGL 582 m@800 m AGL	230 m@300 m AGL	585 m@8 × 5 m 360 m@5 × 5 m 125 m@2.5 × 2.5 m	400 m
Typical Bathymetric Point Spacings	2 × 2 m (Deep) 0.7 m × 0.7 m (Shallow)	1.5 × 1.5 m	2 × 2 m–8 × 5 m	0.2 × 0.2 m–0.8 × 0.8 m
Maximum depth	~60 m 2.5–3× Secchi depth	~27 m 1.5–2.5× Secchi depth	~80 m 2.5–3× Secchi depth	~10 m 1× Secchi depth

## Data Availability

Not applicable.
